# In Silico Screening and Molecular Dynamics Simulation of Potential Anti-Malarial Agents from *Zingiberaceae* as Potential *Plasmodium falciparum* Lactate Dehydrogenase (PfLDH) Enzyme Inhibitors

**DOI:** 10.21315/tlsr2023.34.2.1

**Published:** 2023-07-21

**Authors:** Muhammad Fikri Heikal, Wira Eka Putra, Sustiprijatno, Muhaimin Rifa’i, Arief Hidayatullah, Febby Nurdiya Ningsih, Diana Widiastuti, Adawiyah Suriza Shuib, Baiq Feby Zulfiani, Afrabias Firyal Hanasepti

**Affiliations:** 1 Tropical Medicine International Program, Faculty of Medicine, Khon Kaen University, 123, Mittraparp Highway, Muang District Khon Kaen 40002 Thailand; 2 Biotechnology Study Program, Department of Applied Sciences, Faculty of Mathematics and Natural Sciences, Universitas Negeri Malang, Jl. Cakrawala No.5, Sumbersari, Kec. Lowokwaru, Kota Malang, 65145 East Java. Indonesia; 3 Research Center for Plant Conservation, Botanic Gardens and Forestry, National Research and Innovation Agency, Cibinong-Bogor, West Java, Indonesia; 4 Department of Biology, Faculty of Mathematics and Natural Sciences, Brawijaya University, Jl. Veteran, Ketawanggede, Kec. Lowokwaru, Kota Malang, 65145 East Java, Indonesia; 5 Health Governance Initiative, United Nations Development Programme Indonesia, Eijkman-RSCM Building, Jakarta, Indonesia; 6 Research Center for Vaccine and Drug, National Research and Innovation Agency, South Tangerang, Indonesia; 7 Department of Chemistry, Faculty of Mathematics and Natural Science, Universitas Pakuan, Jl. Pakuan, Tegallega. Kecamatan Bogor Tengah, Kota Bogor, 16143 West Java, Indonesia; 8 Institute of Biological Sciences, Faculty of Science, Universiti Malaya, 50603 Kuala Lumpur, Malaysia

**Keywords:** Anti-Malaria Drug, *In Silico*, Malaria, PfLDH, *Zingiberaceae*

## Abstract

Malaria continues to be a major public health issue in a number of countries, particularly in tropical regions—the emergence of drug-resistant *Plasmodium falciparum* encourages new drug discovery research. The key to *Plasmodium falciparum* survival is energy production up to 100 times greater than other parasites, primarily via the PfLDH. This study targets PfLDH with natural bioactive compounds from the *Zingiberaceae* family through molecular docking and molecular dynamic studies. Sulcanal, quercetin, shogosulfonic acid C, galanal A and naringenin are the Top 5 compounds with a lower binding energy value than chloroquine, which was used as a control in this study. By binding to NADH and substrate binding site residues, the majority of them are expected to inhibit pyruvate conversion to lactate and NAD^+^ regeneration. When compared to sulcanal and control drugs, the molecular dynamics (MD) simulation study indicated that quercetin may be the most stable molecule when interacting with PfLDH.

**Highlights**

- The inhibition of *Plasmodium falciparum* lactate dehydrogenase action as a critical enzyme in the glycolysis may effectively prevent parasite development in the red blood cells stage and possibly decreases malaria pathogenesis.
- Due to bonding into the crucial active site of PfLDH with a higher binding affinity value than control, five bioactive compounds from *Zingiberaceae* plant family were proposed to be a inhibitor candidate, namely sulcanal, quercetin, shogasulfonic acid C, galanal A and naringenin.
- Molecular dynamics simulation analysis demonstrated that quercetin becomes the most stable compound interacting with PfLDH.

## INTRODUCTION

The previous study reported that *Plasmodium falciparum* survival in human red blood cells depends on energy production by elevating glucose consumption during parasite invasion up to 100 fold (Mathema & Na-bangchang 2015; Van Niekerk *et al*. 2016). One of the most critical enzymes that contribute to the energy generation process in *Plasmodium falciparum* is *P. falciparum* lactate dehydrogenase (PfLDH). This enzyme’s role in catalyst conversion pyruvate to lactate generates NAD^+^ from NADH in the glycolytic pathway (Alam *et al*. 2014; Florens *et al*. 2002). PfLDH must generate NAD^+^ to avoid a stall in the glycolysis process (Wirth *et al*. 2018). Inhibiting PfLDH by specific small molecules could theoretically inhibit energy generation during blood-stage infection (Ramakrishnan *et al*. 2017). Also, PfLDH has been used as a biomarker in rapid diagnostic tests due to increased RNA expression during blood-stage infection (Mathema & Na-bangchang 2015). Some research suggested that PfLDH has high structural and functional similarity with other LDH from different *Plasmodium* species, mainly *Plasmodium vivax* (Lee *et al*. 2020; Markwalter *et al*. 2016; Penna-Coutinho *et al*. 2011). Because of its signification in parasite survival and a high degree of similarity with other *Plasmodium* LDH, PfLDH is considered a potent target for this study. In addition, there is still no definitive drug directly focusing in PfLDH on malaria treatment until this day (Markwalter *et al*. 2016). However, one of the most well-known lactate dehydrogenases inhibitor is oxamate. Oxamate inhibits lactate dehydrogenase by binding to the specific binding sites for pyruvate and the NADH cofactor, effectively halting the pyruvate to lactate conversion process (Choi *et al*. 2007; Patrick 2020; Zhao *et al*. 2015).

Several plants are widely used as traditional medicine for some diseases. One popular plant family that is widely used is *Zingiberaceae*. This plant is highly prevalent in tropical areas, like Southeast Asia (Sirirugsa 1999; Zahara *et al*. 2018). *Zingiberaceae* generally contain many secondary metabolites, including alcohol, terpenes, flavonoid and ketone (Zahara *et al*. 2018). Besides having an excellent pharmacological profile, this plant also positively affects human health by maintaining the immune system. Some previous research revealed that *Zingiberaceae* have antimicrobials, antioxidant, anti-inflammation and anti-parasitic activity (Chen *et al*. 2008; El-Sayed & El-Saka 2015; Yao *et al*. 2018). This study predicted some bioactive compounds from *Zingiberaceae* were antimalarial drug candidates. Previous *in silico* studies against the PfLDH are almost wholly focused on repurposing existing drugs rather than exploring new potent compounds from new sources, especially the natural bioactive compounds (Penna-Coutinho *et al*. 2011; Ramakrishnan *et al*. 2017).

*In silico* screening by molecular docking and dynamic simulation has been conducted on several bioactive compounds of 15 plants from the *Zingiberaceae* to explore its potential as potent inhibitors of PfLDH. Molecular docking revealed the interaction between target protein and ligand by analysing binding affinity, type and interaction strength. The molecular dynamic simulation study was performed to confirm docking results by analysing the stability and flexibility of the protein-ligand complex over simulation (Priya *et al*. 2015).

## MATERIAL AND METHODS

### Bioactive Compound Data Collection

The bioactive compounds data of 15 plants from the *Zingiberaceae* family were collected from various literature (Al-Adhroey *et al*. 2010; Amadi *et al*. 2016; Ayimele *et al*. 2004; Chaudhary *et al*. 2018; Chumroenphat *et al*. 2019; Dosoky & Setzer 2018; Duker-Eshun *et al*. 2002; Elshamy *et al*. 2019; Igoli *et al*. 2012; Kenmogne *et al*. 2005; Lategan *et al*. 2009; Ma *et al*. 2017; Mao *et al*. 2019; Nurcholis *et al*. 2012; Sharifi-Rad *et al*. 2017; Sriphana *et al*. 2013; Tane *et al*. 2005; Tchuendem *et al*. 1999; Thongnest *et al*. 2005; Tian *et al*. 2020; Wabo *et al*. 2006; Zofou *et al*. 2013). We use the PubChem website (S. Kim *et al*. 2016) to collect the three-dimensional structure of bioactive compounds in SDF format. Each compound ID number is recorded for validation purposes.

### Lipinski Rule of Five Validation

The 3D structure compounds downloaded from PubChem were tested using the Lipinski rule of five (Lipinski 2004). We set up pH values based on neutral conditions (7). The compounds used for oral therapies are classified as small molecules (Goodwin *et al*. 2017). This test determines whether compounds can be used as oral drugs or not based on physical and chemical properties (Lipinski 2004).

### Protein Target Preparation

The protein used as a target in the present study is PfLDH. PfLDH is a key enzyme in the Plasmodium glycolysis process during red blood cell infection (Alam *et al*. 2014). PfLDH 3D Conformer (PDB ID:1CEQ) downloaded from RSCB PDB (https://www.rscb.org/) in PDB format (Read *et al*. 1999). The protein structure obtained from RSCB PDB still consists of native ligands such as methyl hydrogen carbonate. In this experiment, PyMol v.2.5.2 (https://www.pymol.org) was used to remove the native ligand so the tested compounds could access the target protein’s active sites later in the docking procedure.

### Molecular Docking

The docking process is performed using Autodock Vina (https://pyrx.sourceforge.io/). About 137 selected compounds were minimised and converted into pdbqt format to optimise the docking process. PfLDH protein converted into autodock molecular format (pdbqt) before docking. Chloroquine (CID:2719) was used as a control in this present study. Chloroquine bind to the NADH binding site of PfLDH and becomes a positive inhibitor (Read *et al*. 1999). The grid size used was X: 80.9384, Y:0.5926, Z:50.6613, and coordinate/dimensions (Angstrom) used was X:109.3012, Y:111.0729, Z:111.2643. From 138 selected compounds (natural bioactive compounds and control), Top 5 compounds with the lowest binding affinity value were chosen for the visualisation process.

### Docking Result Visualisation

The Top 5 compounds are visualised using PyMol v.2.5.2 and LigPlot+ v.2.2 (https://www.ebi.ac.uk/thronton-srv/software/LigPlus). PyMol is used for 3D visualisation, while LigPlot+ is for 2D visualisation. The visualisation process aims to see how the bond between PfLDH and tested compounds.

### Molecular Dynamic Simulation

For molecular dynamic simulations against the PfLDH protein, the top two ligands with the lowest binding affinity scores and a drug control were chosen molecular dynamic simulation. The protein and ligand complex structure was prepared and set up according to typical physiological circumstances (37°C, 1 atm, pH 7.4, 0.9% salt content) for 1,000 picosecond simulations. The md run macro programme was used to run the molecular dynamics simulation, and the md analyse and md analyeres macro programmes were used to analysed the molecular dynamics data.

## RESULTS

From 137 natural compounds (Table 1), selected five compounds that have the best binding affinity to PfLDH, including sulcanal (ID: 21604870), quercetin (ID: 5280343), shogasolfunic acid C (ID: 101232217), galanal A (ID: 3050416), and naringenin (ID: 932). These compounds have a lower binding energy value than control, ranging from 24.6%–73.8% lower than chloroquine (−6.1 kcal/mol). The docking result of Top 5 compounds and control is further described in Table 2.

Sulcanal was predicted to have the best binding affinity to PfLDH (−10.6 kcal/mol). Sulcanal bind to distinct residue compared with all compounds. Sulcanal bind to PfLDH on Arg231(A), Ile239(A), Leu242(A), Arg171(A), Try175(A), Try174(A), Pro184(A), Arg185(A), Lys175(A), Ser170(A), Glu238(A) through hydrophobic contacts. Arg171(A) is one of pyruvate binding site residue.

Quercetin was predicted to have the second-lowest binding energy value after sulcanal (−7.8 kcal/mol); this compound bind to binding site residue of pyruvate, including His195. Quercetin also binds to Asn140, His195 which are two crucial NADH binding site residues. Quercetin binds into NADH and pyruvate binding sites through hydrophobic contact interaction.

**Figure 1:**
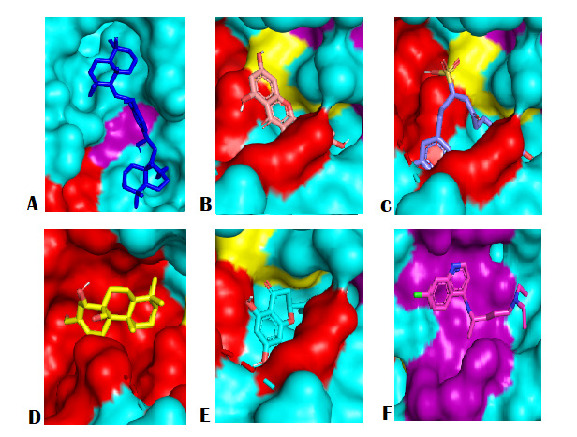
3D visualisation of Top 5 natural compounds and control with PfLDH. (A) sulcanal, (B) quercetin, (C) shogasolfunic C, (D) galanal A, (E) naringenin, and (F) chloroquine (control). Red (Cofactor binding site domain), Purple (Substrate binding site domain), Yellow (Both substrate and cofactor binding site domain).

**Figure 2:**
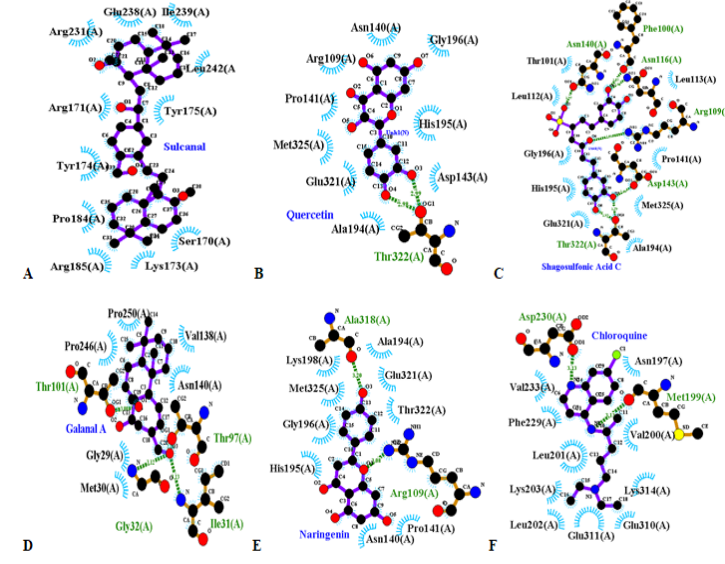
2D visualisation Top 5 compounds and control with PfLDH. (A) sulcanal, (B) quercetin, (C) shogasolfunic C, (D) galanal A, (E) naringenin, and (F) chloroquine (control).

Shogasolfunic acid C was predicted to have the third-lowest binding energy (−7.7 kcal/mol). This compound interacts with three similar residues of quercetin, including His195(A), Asp143(A), and Met325(A). Shogalsholfunic acid C interacts with His195, Phe100 and Asn140, predicting NADH binding pocket residue. Figs. 1 and 2 shows the 2D and 3D visualisation of Top 5 compounds and control with PfLDH.

Galanal A was predicted to have an identical binding energy score with shogasolfunic C (−7.7 kcal/mol). Galanal A interacts with 6 Cofactor binding pocket residues (Asn140, Gly32, Thr97, Met30, Val138, Ile31, Pro250) through hydrophobic contact and H-bond interaction. This compound has some similar binding residue with quercetin, shogasolfunic C and naringenin.

Furthermore, naringenin was predicted to have the fifth-lowest binding energy score (−7.6 kcal/mol). This compound interacts with His195 in the pyruvate binding site through hydrophobic contact interaction. Naringenin bind to Asn140, which is NADH binding site residue. On the other hand, chloroquine as a control has a different binding site compared to all compounds. This drug has the highest binding energy value of the Top 5 compounds (−6.1 kcal/mol). Chloroquine interacts with Val233(A), Asn197(A), Val200(A), Lys314(A), Glu311(A), Leu202(A), Lys203(A), Phe229(A), Leu201(A) through hydrophobic contact, while binding to Asp230(A), Met199(A) through H-bond interaction. Seven chloroquine binding residues are part of the substrate-binding domain, including Val233(A), Val200(A), Lys314(A), Phe229(A), Leu201(A), Asp230(A), and Met199(A).

**Figure 3:**
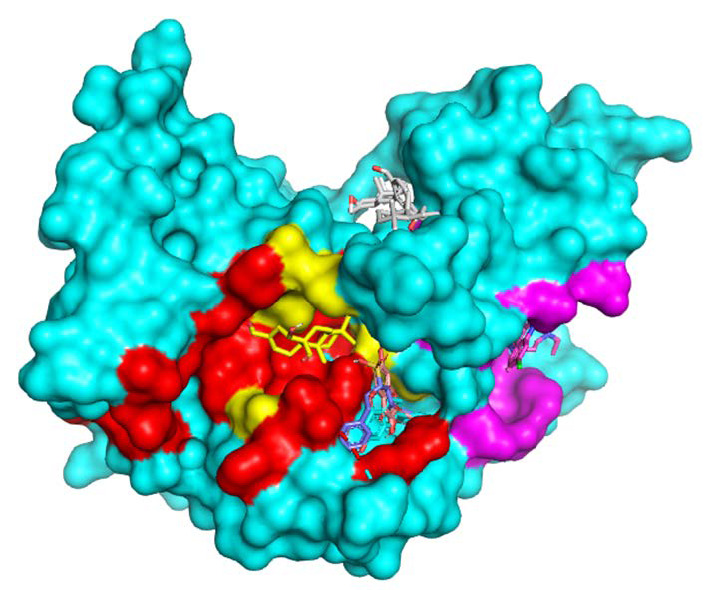
3D visualisation of the ligand-PfLDH complex with binding site explanation. Red (Cofactor binding site domain), Purple (Substrate binding site domain), Yellow (Both substrate and cofactor binding site domain).

The 3D and 2D visualisation results consistently show that chloroquine as control and sulcanal as a compound with the lowest affinity score is localised to an entirely different binding site than the rest of the potent compounds (see Figs. 1–3). Galanal A is also predicted to be almost completely isolated in the separate binding pocket but still share one particular residue with quercetin and naringenin (Asn140). The rest of the potent compounds (quercetin, naringenin and shogasolfunic acid C) are predicted to be localised in the same binding pocket. Those compounds shared about nine conserved residues; they are Arg109, Asn140, Pro141, Ala194, His195, Gly196, Glu321, Thr322 and Met325.

**Figure 4:**
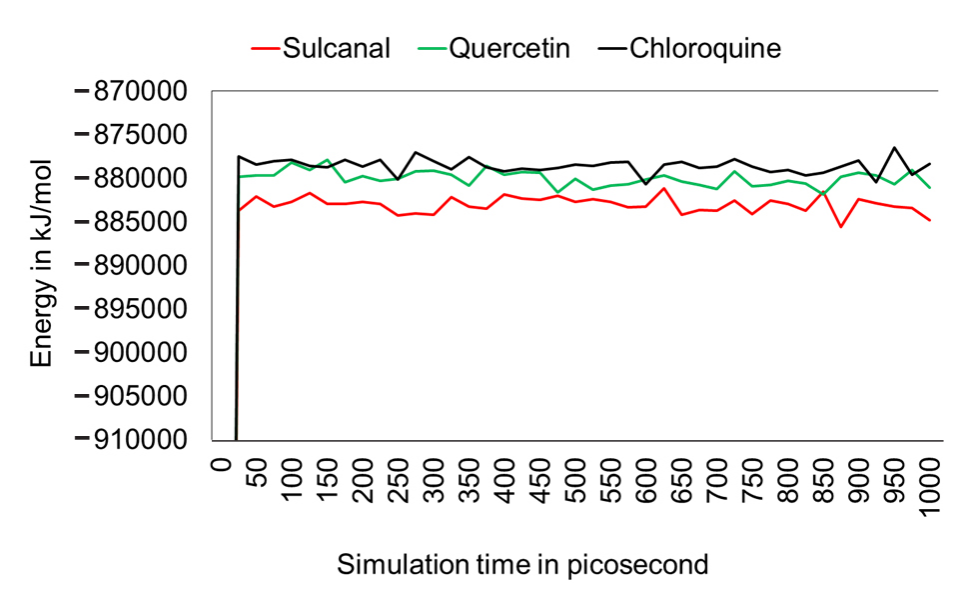
The potential binding energy of sulcanal (red), quercetin (green), and chloroquine (black) during MD simulation over 1,000 picosecond simulations.

After the molecular docking test, the top two compounds (sulcanal and quercetin) and chloroquine as control were analysed together using molecular dynamics (MD) simulation. We analysed three potential parameters, including potential binding energy root mean square deviation (RMSD), root means structure fluctuation (RMSF), solvent accessible surface area (SASA), and rad gyration (Rg) over 1,000 picosecond simulations (see Fig. 4).

**Figure 5:**
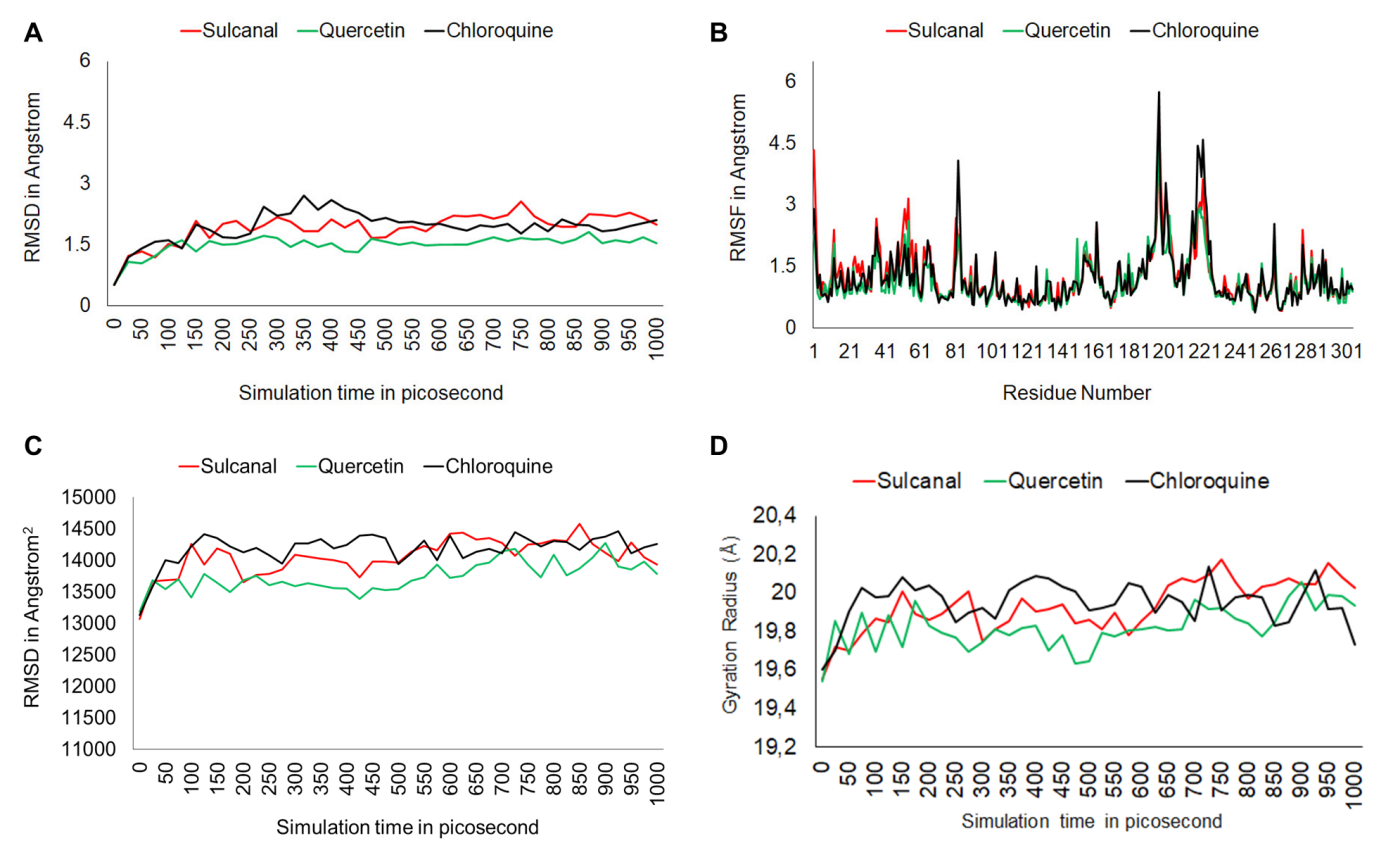
(A) Root mean square deviation graph, (B) Root mean structure fluctuation graph, (C) Solvent accessible surface graphs, and (D) Radius gyration graphs for sulcanal (red), quercetin (green), and chloroquine (black) complex to PfLDH over a 1,000 picosecond of simulation.

The RMSD value PfLDH-sulcanal complex was 1.924 ± 0.374 Å with a maximum of 2.564 Å and a minimum of 0.512 Å. PfLDH-quercetin RMSD value was 1.504 ± 0.218 Å with a maximum of 1.820 Å and a minimum of 0.537 Å. PfLDH-chloroquine complex means the value was 1.942 ± 0.318 Å, ranging from 0.511 to 2.714 Å. PfLDH-quercetin complex has the lowest RMSD value than two other compounds. MD simulation results indicate PfLDH-quercetin is more stable than PfLDH-sulcanal and PfLDH-chloroquine complex over simulation (Fig. 5A).

RMSF analysis result (Fig. 5B) shows that PfLDH-chloroquine complex has a higher RMSF value than two other compounds, indicating that PfLDH-chloroquine is most unstable. However, there are no significant differences between the three compounds. All three compounds have low values for PfLDH residues.

The SASA plot (Fig. 5C) revealed that the three protein-ligand complexes do not undergo expanded over 1,000 picosecond simulations. All three complexes have similar graph patterns before the first 50 ps. The graphs value of all protein-ligand complexes is generally flat. Sulcanal-PfLDH complex reaches maximum SASA value (14500 Å^2^) at 850 picoseconds. SASA analysis result indicates none of the three complexes increase in SASA value over simulation.

The Rg graph (Fig. 5D) shows that none of all three complexes performs a horizontal graphic pattern. Sulcanal, quercetin and chloroquine average score was 19.931 Å, 19.822 Å, and 19.951 Å, respectively. The quercetin-PfLDH complex has the lowest minimum Rg value (19.541 Å), while the sulcanal-PfLDH value has the highest maximum score (20.176 Å).

## DISCUSSION

The PfLDH contributes to the glycolysis process during red blood cell (RBC) invasion. LDH generates NAD^+^ through conversion pyruvate to lactate reaction (Alam *et al*. 2014; Florens *et al*. 2002). NAD^+^ is essential for glycolysis as a cofactor in several glycolytic enzymes (Palmeira & Rolo 2014). This protein is highly expressed during elevated glucose uptake and stops when parasites reach the schizont stage (Jain *et al*. 2014). Besides the potential drug target, this protein has long been a biomarker in malaria rapid diagnostic tests (Mathema & Nabangchang 2015). High lactate concentration can be used in parasitemia tests on infected RBC (Alam *et al*. 2014). *Plasmodium falciparum* LDH has different properties from human LDH. PfLDH is not inhibited when pyruvate is elevated, while human LDH is inhibited on high pyruvate concentrations (Vander Jagt *et al*. 1990).

PfLDH has two regions with different conformational residues with human LDH, including antigenic and substrate specificity loops (Alam *et al*. 2014). Some previous *in silico* studies explain that some potential inhibitors contact several crucial binding pocket residues of PfLDH (Nasution *et al*. 2016; Penna-Coutinho *et al*. 2011). Some drugs such as atorvastatin, itraconazole and posaconazole bind to NADH binding site residues (Penna-Coutinho *et al*. 2011). Some bioactive natural products from *Dioscorea bulbifera* L. also reported high binding affinity to PfLDH, such as 2,4, 3′,5′-tetrahydroxybibenzyl, and quercetin (Chaniad *et al*. 2021). These compounds were then tested using *in vitro* study and revealed that quercetin gives the most potent antimalarial effect to *Plasmodium falciparum* K1 and 3D7 strains (Chaniad *et al*. 2021).

The docking result shows that five bioactive compounds have a higher binding affinity than chloroquine (control), and some compounds are incorporated in the adjacent regions, except sulcanal and chloroquine. The Top 5 compounds have physical and chemical properties according to Lipinski’s rule of 5. We predict these compounds possess pharmacokinetic capabilities as oral drugs. The Lipinski rule of five validation data is shown in Table 3.

Sulcanal is a diterpenoid founded in *Aframommum latifolium* (Duker-Eshun *et al*. 2002). This compound has the lowest binding energy score to PfLDH through hydrophobic contact. Seven sulcanal binding site residues, including Arg231(A), Ile239(A), Arg171(A), Pro184(A), Arg185(A), Ser170(A), and Glu238(A), are similar with 1,2,4,5-tetraoxane-8-aminoquinoline hybrids binding pocket in PfLDH that tested by the similar study before (Mahmud *et al*. 2020). Sulcanal bind Arg171(A) as essential pyruvate binding residue. This hydrophobic contact between sulcanal and Arg171 is predicted to interfere with pyruvate binding to PfLDH.

The following compound is quercetin, a flavonoid found in *Curcuma longa* (Chinedum *et al*. 2015; D. W. Kim *et al*. 2016). Furthermore, this compound is also present in onion, apples, honey, red grapes, cherries and green leafy vegetables (Hollman & Katan 1999). Quercetin has antimicrobe, anti-inflammatory, antioxidant, and antifungal activity (Jaisinghani 2017; Saeedi-Boroujeni & Mahmoudian-Sani 2021; Singh *et al*. 2015; Xu *et al*. 2019). *In vitro* study revealed that quercetin has a significant antimalarial effect on *Plasmodium falciparum* (Chaniad *et al*. 2021). In this study, quercetin has the second-highest binding affinity to PfLDH. Quercetin binds to 2 residues of NADH (Asn140, His195) through hydrophobic contact. His 195 is also a residue of pyruvate. There is a possibility that the binding quercetin to the crucial residues of PfLDH would inhibit pyruvate attachment and prevent NAD^+^ regeneration. The type of interaction included in the quercetin-PfLDH complex is hydrophobic contact and H-bond interaction. Hydrogen bond distances between O3 and O4 to Thr322(A) are equal to 2.99 and 2.98 Å, respectively. These H-bond involved moderate interaction.

Shogasolfunic C and Galanal A is the third-highest binding affinity value. Shogalsulfonic acid C originated from *Zingiber officinale*, while Galanal A present in the fruit of *Aframommum latifolium* (Duker-Eshun *et al*. 2002; Hori *et al*. 2003). Shogasolfunic C and Galanal A have identical binding residues, including Thr101(A) and Asn140(A). Both compounds bind in cofactor critical site residue through hydrophobic contact and H-bond interaction. These compounds could be potential inhibitors to PfLDH by preventing NAD^+^ regeneration. Three cofactor binding residues in galanal A-PfLDH (Gly32, Thr97, Ile31) complex involved in H-bond interaction with moderate distance. It indicates there is a possibility of bond instability between the atoms to these residues. Shagosulfonic Acid C also interacts with two crucial cofactor residues (Asn140, Phe100) through H-bond interaction. O3 atom in sulfonate group bind to Asn140 with a distance of 2.97 Å. On the other hand, Phe100 bind to O4 with an H-bond distance equal to 2.87 Å. Both H-bond distance values indicate moderate interaction.

The last potential compound as an antimalarial drug candidate is naringenin. Naringenin has the highest binding energy score compared to 4 natural compounds (−7.6 kcal/mol). Naringenin bind to 1 pyruvate and NADH binding site residue through hydrophobic interaction. This compound is possible to be a PfLDH inhibitor by disturbing substrate and cofactor attachment. In a previous study, naringenin has a hepatoprotective ability that protects the liver from damage in many diseases (Hernández-Aquino & Muriel 2018). Naringenin also has anticancer, anti-inflammatory, antioxidant, and antifibrogenic activity (Hernández-Aquino & Muriel 2018). In vitro study revealed that flavones compounds, including naringenin, show antiplasmodial action against *Plasmodium falciparum* chloroquine-sensitive (3D7) and chloroquine-resistant (7G8) (Lehane & Saliba 2008).

The Top 5 also compounds chloroquine exhibit hydrophobic contact and hydrogen bond to bind on the protein target. Hydrophobic contact has a more substantial bonding effect than any noncovalent bond such as hydrogen and *van der Waals* interaction (Atkins & Paula 2010; Berg *et al*. 2002). Thus, hydrophobic contact contributes to most biological processes, significantly facilitating drug delivery through the cell membrane and protein-protein recognition (Dutta *et al*. 2014; Berg *et al*. 2002). The chemical structure of theTop 5 compounds may help their delivery process pass through the cell membrane until PfLDH is located in cytosol and cytoplasm. In addition, increasing the number of hydrophobic atoms in the active core of the drug-target interface could increase the drug candidate’s biological activity. However, the drug’s binding affinity and efficacy can be optimised by incorporating hydrophobic interactions at the hydrogen bonding site (Varma *et al*. 2010).

Interestingly, three of the Top 5 compounds predicted to have an antiplasmodial effect are included in *Aframommum latifolium*. Sulcanal, galanal A and naringenin are included in *Aframommum latifolium*, which are suggested to potentially act as antiplasmodial (Amadi *et al*. 2016). Both sulcanal and galanal A are categorised as labdane diterpenoid. Most labdanes diterpenoid has been known for years due to its bioactivity as antiplasmodial, especially in chloroquine-resistant *P. falciparum* cases (Endale *et al*. 2013). The chemical structure of labdane diterpenoid may give influence its activity as antiplasmodial. Although it still needs further investigation.

The Top 5 compounds are considered a novel antiplasmodial drugs candidate; three binds to NADH and substrate binding site residue, one compound only binds to NADH binding site residue, and another binds to hybrid form current antimalarial drug (Aminoquilonine) residues. These compounds have a lower binding affinity value than chloroquine as a control. Bonding with critical active site residue suggests that pyruvate conversion to lactate and NAD^+^ regeneration may be inhibited. It can hinder the malaria parasite’s energy production, especially in the merozoite and trophozoite developmental stages. Inhibition development of merozoite and trophozoite is related to many aspects of malaria pathogenesis. When merozoite development is inhibited, gametocyte will not develop because it is formed from merozoite progeny (Ngwa *et al*. 2016). Trophozoite stage related to three crucial protein expressions that mediated rosetting phenomena in falciparum malaria, including PfEMP1, RIFIN and STEVOR (Yam *et al*. 2017). Inhibition of this stage may reduce severe malaria pathogenesis.

The molecules undergo atom motion play in ligand binding (Choi *et al*. 2018). Therefore, *in silico* analysis using the molecular docking method is not enough to represent the reliable effectiveness of the drug to target protein. We tested the Top 2 bioactive compounds and a control drug by the molecular dynamic simulation to optimise the virtual screening of *Zingiberaceae* compounds to a target protein. The result shows that the quercetin-PfLDH complex has the lowest RMSD value compared to the chloroquine and sulcanal complex to PfLDH. That indicates quercetin has the most stable interaction to PfLDH during simulation than two other compounds. In RMSF analysis, we conclude that chloroquine shows the highest RMSF value and indicates this compound has the most unstable interaction with PfLDH amino acid residues than others. We also performed SASA and Rg analysis. SASA analysis is used to explain bimolecular surface area that is assessable to solvent molecules (Kamaraj & Purohit 2013). Three complexes initially slightly increased SASA value and then performed a flat trend. We concluded that there is no tendency to shrink and expand the surface of the protein-ligand complexes. Radius gyration was used to measure the overall size of a chain molecule and represent conformational protein changes during simulation (Jiang *et al*. 2019). Results indicate sulcanal-PfLDH and quercetin-PfLDH complexes are more compact in conformation than chloroquine-PfLDH due to having a lower Rg value.

## CONCLUSION

Five bioactive compounds from the *Zingiberaceae* plant family were proposed to be a potential inhibitor to PfLDH due to bonding into the crucial active site of PfLDH with a higher binding affinity value than control. The inhibition of PfLDH action as a critical enzyme in the glycolysis process may effectively prevent parasite development in the RBC stage, especially in merozoite and trophozoite. Inhibition of merozoite and trophozoite possibly decreases malaria pathogenesis. Based on MD simulation analysis, it suggested that quercetin becomes the most stable compound interacting with PfLDH.

## Figures and Tables

**Table 1:** Docking result of all compounds towards PfLDH protein.

No.	Plant	Compound
1	*Zingiber officinale*	10-gingerol; 2-tetradecanol; 2-pinen-5-ol; 2,6-dimethylhept-5-enal; 2-decanol; 2-nonanol; 2-undecanone; 4-isopropylbenzyl alcohol; 4-gingesulfonic acid; 6-gingerol; 6-gingesulfonic acid; 8-gingerol; Cis-piperitol; Cis-linalool oxide; Cis-sesquisabinene hydrate; Citral; Citronellol; Dihydrocurcumin; Elemol; Foliamenthic acid; Geranic acid; Geranyl propionate; Gingerenone A; Hexahydro curcumin; Isoborneol; Isobornyl formate; Myrtenol; Paradol; Shogaol; Taumuurolol; Terpinolene; trans-Sabinene hydrate; Undecanoic acid; Zingerone; Zingiberene
2	*Zingiber zerumbet*	1,8-cineole; 4-terpeniol; 6-dehydrogin gerdione; 6-gingerol diacetate; Allo-aromadendrene epoxide; Bornyl acetae; Camphene; Camphor; Caryophyllene oxide; D-nedoridol; Fenchone; Hedycaryol; Humulene oxide I; Humulene oxide II; Linalol; Myrtenyl Acetate; P-cymene; Sabinene; Shogasulfonic Acid A; Shogasulfonic Acid B; Shogasulfonic Acid C; Shogasulfonic acid D; Tricyclene; Verbenone; Zerumbone; α-pinene
3	*Curcuma longa*	Ar-turmerone; Caffeic acid; Chlorogenic acid; Cinnamic acid; Coumarin; Curcumin; Curlone; Curzerenone; Demethoxy curcumin; Epicatechin; Ferulic acid; Genistein; Germacrone; Ledane; Myrcene; Myricetin; Quercetin; Sinapic acid; Syringic acid; Vanillic acid
4	*Kaempferia galanga*	1,6-cyclodecadiene; 3-carene; 3-caren-5-one; Alpha Gurjunene; Alpha Terpineol; Borneol; Cadinene; Carvone; Cinnamaldehyde; Ethyl cinnamate; Gamma elemene; Kaempferide; Tetradecane
5	*Alpinia galanga*	1,7-bis (4-hydroxyphenyl) −1,4,6-heptatrien-3-one; 1′-acetoxychavicol acetate; 1′S-1′-acetoxyeugenol acetate; 3-O-acetyl pinobanksin; 4-hydroxyben zaldehyde; Alpinone; Di-(p-hydroxy-cis-styryl) methane; Galanganol B; Galangin; Isocoronarin D; Pinobanksin
6	*Aframomum latifolium*	Aframodial; Coronarin B; Galanal A; Galanal B; Naringenin; Sulcanal
7	Boesenbergia rotunda	Alpinetin; Cardamonin; Hemanthidine; Kaempferol; Limonene; Pinocembrin
8	*Kaempferia rotunda*	5-Hydroxy-7-methoxyflavanone; 5,7-dihydroxyflavanone; Crotepoxide; Zeylenol
9	*Aframomum zambesiacum*	3-deoxyaulac ocarpin A; Aulacocarpine A; Zambesiac olactone A; Zambesiac olactone B
10	*Aframomum arundiaceum*	6,7-epoxy-3(15) - caryophyllene; Alpha-bisabolol; Galanolactone
11	*Siphonochilus aethiopicus*	Epi-curzerenone; Germacrene D; Zerumin A
12	*Curcuma zanthorriza*	Bisdemetho xycurcumin; Xanthorrizol
13	*Aframomum escapum*	S-nerolidol
14	*Siphonochilus aethiopicus*	Furanodiene
15	*Renealmia cincinnata*	Oplodiol

**Table 2:** Description of docking result of Top 5 compounds with the binding site residue. Bold indicates NADH binding site residue, and the underline indicates substrate-binding site residue.

No	Compound	Binding affinity	Amino acid residue	Interaction (Å)
1	Sulcanal (ID:21604870) Source: *Aframommum latifolium*	−10.6 (kcal/mol)	Arg231(A), Ile239(A), Leu242(A), Arg171(A), Try175(A), Try174(A), Pro184(A), Arg185(A), Lys175(A), Ser170(A), Glu238(A)	Hydrophobic contact
2	Quercetin (ID:5280343) Source: *Curcuma longa*	−7.8 (kcal/mol)	Arg109(A), Gly196(A), **His195(A)**, Glu321(A), Ala194(A), **Asn140(A)**, Pro141(A), Met325(A), Asp143(A)	Hydrophobic contact
			Thr322(A)	Hydrogen bond (2.99) (2.98)
3	Shogasulfonic Acid C (ID:101232217) Source: *Zingiber officinale*	−7.7 (kcal/mol)	Thr101(A), Leu112(A), Gly196(A), **His195(A)**, Glu321(A), Met325(A), Pro141(A), Leu113(A), Ala194(A)	Hydrophobic contact
			Thr322(A)	Hydrogen bond (3.92) (2.79)
			Asp143(A)	Hydrogen bond (3.09)
			Arg109(A)	Hydrogen bond (2.89)
			Asn116(A)	Hydrogen bond (2.98)
			**Phe100(A)**	Hydrogen bond (2.87)
			**Asn140(A)**	Hydrogen bond (2.97)
4	Galanal A (ID: 3050416) Source: *Aframommum latifolium*	−7.7 (kcal/mol)	**Pro250(A)**, Pro246(A), **Val138(A)**, **Asn140(A)**, Gly29(A), **Met30(A)**	Hydrophobic contact
			Thr101(A)	Hydrogen bond (3.08)
			**Gly32(A)**	Hydrogen bond (3.12)
			**Thr97(A)**	Hydrogen bond (3.15)
			**Ile31(A)**	Hydrogen bond (3.13)
5	Naringenin (ID: 932) Source *Aframommum latifolium*	−7.6 (kcal/mol)	Lys198(A), Met325(A), Gly196(A), **His195(A)**, **Asn140(A)**, Pro141(A), Ala194(A), Thr322(A), Glu321(A)	Hydrophobic contact
			Ala318(A)	Hydrogen bond (3.20)
			Arg109(A)	Hydrogen bond (3.08)
6	Chloroquine (ID: 2719) Control/Drug	−6.1 (kcal/mol)	Val233(A), Asn197(A), Val200(A), Lys314(A), Glu311(A), Leu202(A), Lys203(A), Phe229(A), Leu201(A)	Hydrophobic contact
			Asp230(A), Met199(A)	Hydrogen bond (3.13)

**Table 3:** Top 5 Lipinski rule of five validation data.

Compound	Mass (Dalton)	LogP	H-bond donor	H-bond acceptor	Molar reactivity
Sulcanal CID : 21604870	312	−0.053	5	6	77.14
Quercetin CID : 5280343	302	2.010	5	7	74.05
Shogasulfonic Acid C CID : 101232217	410	3.707	5	8	101.18
Galanal A CID : 3050416	318	3.694	1	3	89.83
Naringenin CID : 932	272	2.509	3	5	70.19
